# Effectiveness of Partial Body Weight-Supported Treadmill Training on Various Outcomes in Different Contexts among Children and Adolescents with Cerebral Palsy: A Systematic Review and Meta-Analysis

**DOI:** 10.3390/children11010009

**Published:** 2023-12-20

**Authors:** Abdulmajeed Alotaibi, Alaa Ibrahim, Raafat Ahmed, Turki Abualait

**Affiliations:** 1 Department of Physical Therapy, Children’s Hospital, Ministry of Health, Taif 26514, Saudi Arabia; 2 Department of Physical Therapy, College of Applied Medical Sciences, Imam Abdulrahman Bin Faisal University, Dammam 34212, Saudi Arabia; aiibrahim@iau.edu.sa (A.I.); rmahmed@iau.edu.sa (R.A.); tsabualait@iau.edu.sa (T.A.)

**Keywords:** cerebral palsy, treadmill, children, adolescent, randomized controlled trial, rehabilitation, home, school

## Abstract

The efficiency of partial body weight-supported treadmill training (PBWSTT) for treating various conditions in children and adolescents with cerebral palsy (CP) in diverse contexts of rehabilitation, households, or schools is unknown. The major objective of this systematic review and meta-analysis was to analyze the effectiveness of PBWSTT on various outcomes in different contexts among children and adolescents with CP. We incorporated full-text, randomized controlled trial studies that specifically assessed the effects of PBWSTT walking, motor function, stride, balance, and endurance in children and adolescents aged 3 to 18 years with CP. The literature search was carried out using Google Scholar, PubMed, Web of Science, CINAHL Plus, Scopus, PEDro, and ResearchGate databases. The methodological quality was evaluated using a Cochrane risk of bias instrument. A meta-analysis of pooled data from 10 studies with 255 participants demonstrated that PBWSTT for 4–12 weeks in rehabilitation (mean difference [MD] = 1.94, 95% confidence interval [CI] = 1.40—2.48, *p* < 0.0001), at home or in a school context (MD = 13.5, 95% CI = 13.9—16.0, *p* < 0.0001), was significantly effective for treating various conditions in children and adolescents suffering with CP. The period of 4–12 weeks of PBWSTT in rehabilitation and at-home/school settings is effective on various outcomes in children or adolescents with CP.

## 1. Introduction

Cerebral palsy (CP) is a significant global health issue that affects between one and four of every thousand live births worldwide [1,2]. In the United States of America, approximately 2.4 of every 1000 children are affected by CP [3]. Additionally, the prevalence of CP can vary among males and females. For instance, males generally experience a higher prevalence of CP compared to females, with males in Europe having a rate that is 1.3 times higher [4]. The occurrence of CP among Arabic-speaking countries has been documented as 1.8 cases per 1000 live births [5,6]. For example, Saudi Arabia has one of the lowest rates of CP at 1.6 per 1000 live births [5], although this is still higher than the global average [7].

While there is currently no known cure for CP, certain supportive therapies, such as physical [8] and occupational therapy [9], can help partially manage it, specifically treadmill training [10]. Treadmill training has recently drawn attention as a helpful gait training strategy, in addition to traditional methods, and has many benefits [11]. Partial body weight-supported treadmill training, abbreviated as PBWSTT, is a specific type of training where a person is supported by a harness or other supportive device that reduces the amount of weight they bear while walking on the treadmill [12]. It has been demonstrated that partial body weight-supported treadmill training is useful in helping people with illnesses of a neurological nature, such as spinal cord injury, brain injury, or stroke, to regain their balance, walking speed, and walking endurance [13,14].

Several previous studies [11,15,16] have reported that PBWSTT is safe and effective in treating CP in children across various age groups and conditions. For example, PBWSTT is safe and practical for children with CP, according to a prior review [11], and it may even improve their overall gross motor abilities and walking speed over short distances. According to data from a previous Cochrane systematic review [15], PBWSTT could accelerate the development of independent walking in children with Down syndrome who are younger than six years old. According to a prior randomized controlled trial [16], it is safe and practicable to administer nine weeks of twice-weekly PBWSTT in a special school setting to increase walking speed in children with CP who are ten years of age or older and have moderate to severe walking difficulties.

Considering the benefits and the task-specific nature of PBWSTT, it is unknown whether this intervention may have a similar impact on various outcomes of same-conditioned children aged 3–18 years. Additionally, it is worth exploring whether PBWSTT would yield different results in other settings, such as rehabilitation, home, or school environments, for this group. Therefore, our objective was to review the current scientific data on the efficacy of PBWSTT on various outcomes among children with CP in various settings. This study hypothesized that PBWSTT could potentially benefit, regardless of the specific context, children aged 3 to 18 years with CP.

## 2. Materials and Methods

We followed the recommendations and checklists of the Preferred Reporting Items for Systematic Reviews and Meta-Analyses, abbreviated as PRISMA [17], for the development of the review [18]. The Cochrane Collaboration’s recommendations were followed for conducting a literature review, applying selection criteria, extracting data, and performing statistical analysis [19]. This study, as a literature review, is exempted from Institutional Review Board approval. The registration of this systematic review has been documented in PROSPERO, with the number code CRD42023405443.

### 2.1. Search Strategy

Systematic searches of randomized controlled trial studies were conducted on the Web of Science, PubMed, CINAHL Plus, Google Scholar, the Physiotherapy Evidence Database (PEDro), Scopus, and ResearchGate up to 28 September 2022. These databases were specifically selected for this review based on their reputations as professional databases relating to rehabilitation and physiotherapy. Search terms included specific terms, such as “children”, “young”, “adolescent” “cerebral palsy”, “cerebral palsies”, “infantile palsies”, “treadmill training”, “partial body weight treadmill”, “body weight-supported training”, “treadmill therapy”, “partial body weight treadmill”, and “PBWSTT.” Before reading the complete text, articles were initially chosen based on their keywords, and then on their titles and abstracts.

### 2.2. Eligibility Criteria

The selection criteria for inclusion in this study encompassed articles composed in the English language, with all of them utilizing randomized controlled trials. Studies comprising children and teenagers with CP aged 3 to 18 years were included. Trials were eligible for inclusion that were available in full text, used PBWSTT as the exposure, and its effectiveness on various outcomes (such as walking, motor function, stride, balance, endurance, etc.), in different settings as an outcome. We excluded studies that used combined interventions (treadmill and other therapy) and where the outcome data were not clear.

### 2.3. Data Extraction

Based on the data given in the abstracts and the titles of the studies, we meticulously reviewed and removed any information that appeared to be irrelevant. Afterwards, we carefully examined the complete content of the remaining articles and compared them against the predetermined criteria for eligibility to individually identify and choose the pertinent ones. Subsequently, the final set of eligible studies was chosen based on specific inclusion/exclusion criteria. In this meta-analysis, we focused on incorporating previous studies that reported the mean and ninety-five percent confidence interval (95% CI) to assess the efficiency of PBWSTT on various outcomes in different settings.

### 2.4. Assessment of Methodological Quality

We assessed the methodology of each included study independently, employing the PEDro scale. This particular scale was selected because of its utilization in clinical trials and its important role in assessing the methodological components of such trials. It has also been used in rehabilitative therapies and for restorative purposes and results. Study quality is rated using the PEDro scale, which has a scale from 0 to 10. The experts decided on the PEDro score elsewhere [4], classifying it as 0–3 for poor, 4–5 for fair, 6–8 for good, and 9–10 for excellent. The PEDro score has shown “fair” to “excellent” inter-rater reliability (ICC, Intraclass Correlation Coefficient = 0.53–0.91) for clinical trials. Similarly, this reliability for the specific items of the PEDro scale in physiotherapy trials varies from “fair” to “almost perfect” (k = 0.36–1.00). The PEDro scale’s validity and reliability have been documented in the past [20].

### 2.5. Assessment of the Risk of Bias

We employed the Cochrane Collaboration tool to evaluate bias risk in each of the randomized controlled trials [21]. The tool is structured with a predefined collection of bias categories that focus on different aspects of trial planning, implementation, and reporting. Each evaluation conducted using the tool focuses on a particular randomized controlled trial result. A set of inquiries (designated as “signaling questions”) within each domain tries to extract details regarding characteristic features of the trial that are relevant to the risk of bias. Using the feedback to the signaling queries, an algorithm makes recommendations about the potential bias risk associated with each domain. The algorithm classifies the bias risk as either “High”, “Low”, or “Unclear” [21]. The Cochrane Collaboration tool’s validity and reliability were fair [22].

### 2.6. Statistical Analysis

We looked at multivariate-adjusted data on various outcomes, presented as a mean and 95% CI, to assess the effectiveness of PBWSTT in any setting (primary outcome) and its differences in various contexts (secondary outcome) among patients with CP. The risk of bias summary and risk of bias graph are displayed in traffic light form. The fixed-effect model combined the findings if there was additional relevant study heterogeneity. In other situations, a random-effects model was utilized. The values were shown as mean difference (MD) and 95% CI. The I^2^ statistic was utilized for assessing heterogeneity. The degree of study heterogeneity was classified as moderate (30–59%), significant (60–89%), or great (90–100%) [23]. Funnel plots were employed to assess the likelihood of bias of publication affecting both the primary and secondary outcomes [24]. All data analyses were carried out using RevMan version 5.4 software (the Cochrane Collaboration, Denmark) for Windows. Statistical significance was determined as *p* < 0.05, a *p*-value below 0.05.

## 3. Results

### 3.1. Systematic Review

**Figure 1:**
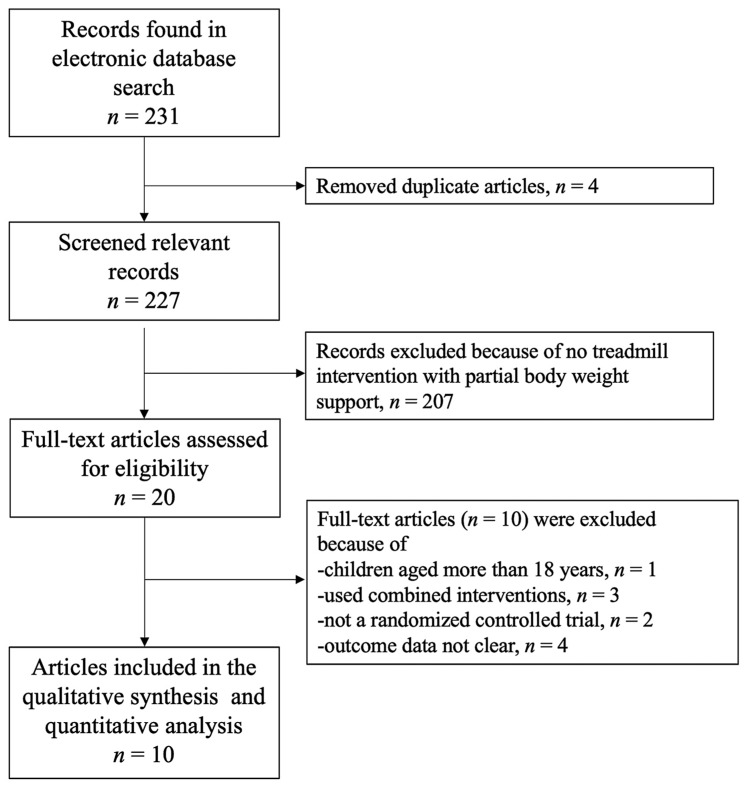
The flow of articles identified in the electronic database search.

A pool of 232 records was found after database searches (Figure 1). The abstracts and titles of these articles were reviewed. After a manual recheck and deduplication, 227 main titles were reviewed, and 207 of them were eliminated. Figure 1 presents the reason for the elimination. Of twenty studies, ten studies (four in rehabs, four in schools, and two with at-home settings) [3,16,25,26,27,28,29,30,31,32] with 255 children were found to be potentially appropriate for the review and included in a full-text review for qualitative synthesis and quantitative analysis. The remaining 10 studies were disqualified for a variety of reasons, including combination therapies, age over 18 years, lack of randomized control trial research, and unclear outcome data.

### 3.2. Quality and Risk of Bias

**Figure 2:**
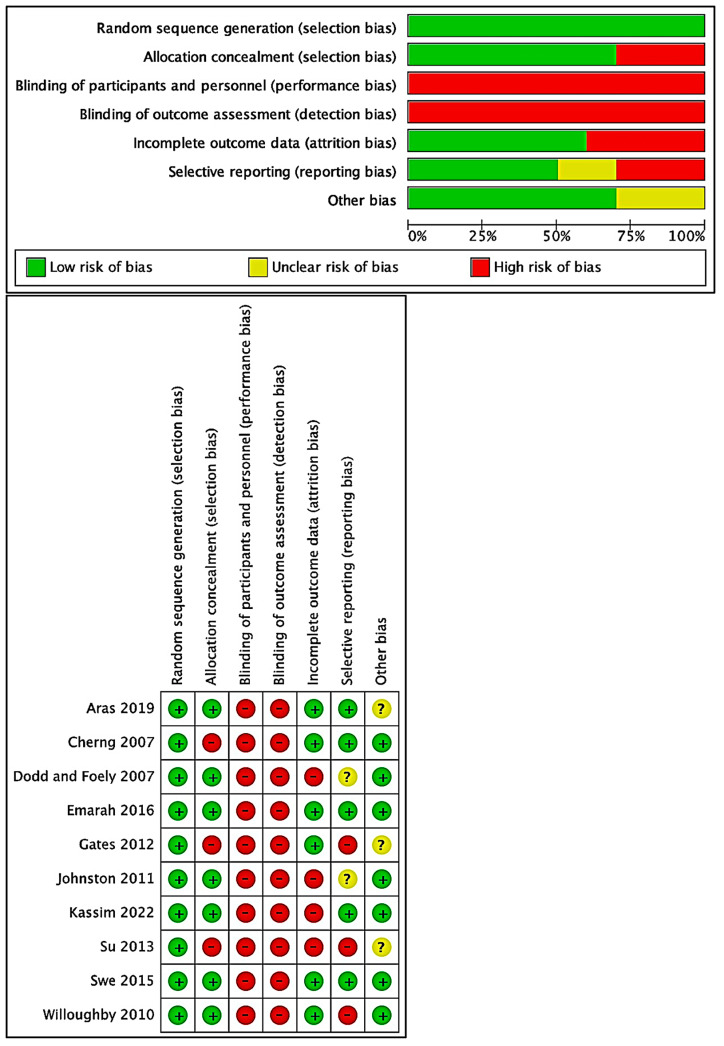
Risk of bias of all included randomized controlled trials. Positive symbols denote low bias risk, negative symbols indicate high bias risk, and question marks suggest unclear for bias risk [3,16,25,26,27,28,29,30,31,32].

The articles selected for analysis [3,16,25,26,27,28,29,30,31,32] received PEDro values ranging from 3 to 8, along with 6 median scores, indicating articles of good quality. The risk of bias in the selected studies [3,16,25,26,27,28,29,30,31,32] is shown in Figure 2. Every single one of these studies received a 100% rating for low-risk reporting and selection biases. All of these studies, however, had at least one element that was highly susceptible to bias. Because the participant, assessor, and therapist were not blinded to treatment allocation, there is a 100% chance of performance and detection biases in all these included studies. A substantial risk of attrition bias (40%) exists in four studies [3,29,30,32] because of insufficient follow-up or data. Due to insufficient outcome data, three studies [3,16,26] were assigned a high risk of reporting bias (30%). All five studies [3,26,28,29,30], however, received a 50% rating for unexplained additional biases.

### 3.3. Characteristic Features of the Included Studies

Characteristics of the included studies are shown in Table 1. In total, 255 participants (38.4% boys), with an average age of 9 years, were split into intervention (*n* = 115) or control (*n* = 100) groups at random, in a rehab center, a home, or a school, for the 10 articles that were included. The sample size of all included studies (*n* = 10) was smaller than the required size [33]. Most of the patients had a mean age of 9 years and had spastic-type CP. The majority of study settings were rehabilitation (*n* = 4) and school (*n* = 4), followed by home (*n* = 2). Most studies were carried out in Australia (*n* = 3), followed by the United States (*n* = 2).

The details of the intervention, treatment time, assessment, outcome variables, and summary of findings have been presented in Table 2. Most of the individuals in the experimental group underwent training sessions, on average lasting 20 to 30 min in the beginning from 4 to 12 weeks and 12 to 24 weeks. The findings from most studies [3,25,26,27,28,30,31,32] show that 4–12 weeks of PBWSTT is significantly effective at improving walking speed, gross motor functions, stability, and balance in various contexts for children with CP. However, two studies [16,29] show there was no significant difference between PBWSTT and exercise in improving gait speed, cadence, or endurance for children with CP in a home or school environment.

Specifically, three studies [28,30,31] that used PBWSTT therapies showed an improvement in walking speed in CP patients. In a Turkish rehabilitation center, one intervention [28] consisted of twenty 45 min sessions held twice a week for four weeks. In an Australian school context, the other intervention [30] involved a walking test lasting 10 weeks. An investigation [31] revealed that after 36 sessions of PBWSTT, i.e., three times a week for thirty minutes for 12 weeks, in the Saudi Arabian rehabilitation environment, children with CP could safely and effectively walk. Following PBWSTT, children with CP had improved gross motor capabilities in three investigations [3,25,27]. One of these three trials used PBWSTT for 20 min per week for 12 weeks in a Taiwanese rehabilitation environment, involving two or three sessions [25]. In a Chinese school, another investigation [3] ran for 262 min throughout 14 PBWSTT sessions over 12 weeks. In an Australian school context, the study conducted two 30 min PBWSTT sessions every week for eight weeks [27].

### 3.4. Meta-Analyses

**Figure 3:**
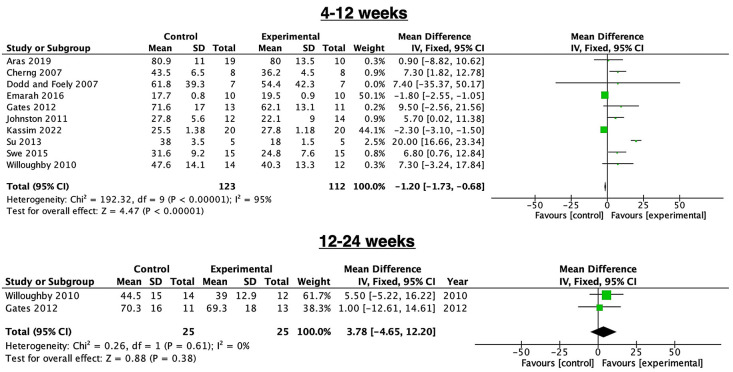
The overall outcome of PBWSTT at 4–12 weeks [3,16,25,26,27,28,29,30,31,32] and 12–24 [16,26] weeks in patients with cerebral palsy. The colored squares represent the odds ratio.

Figure 3 illustrates the effectiveness of PBWSTT for 4 to12 weeks in patients aged between 3 and 18 years with CP. A total of 10 studies [3,16,25,26,27,28,29,30,31,32], involving 255 children, reported that PBWSTT is significantly effective, regardless of setting (MD = −1.20, 95% CI = −1.73, −0.68). However, there was notable heterogeneity covering all studies (I^2^ = 95%, *p* < 0.0001). Two studies [16,26], involving 50 children, showed that PBWSTT for 12 to 24 weeks was not significantly effective (MD = 3.78, 95% CI = −4.65—12.2). There was no important notable heterogeneity between studies (I^2^ = 0%, *p* = 0.61).

**Figure 4:**
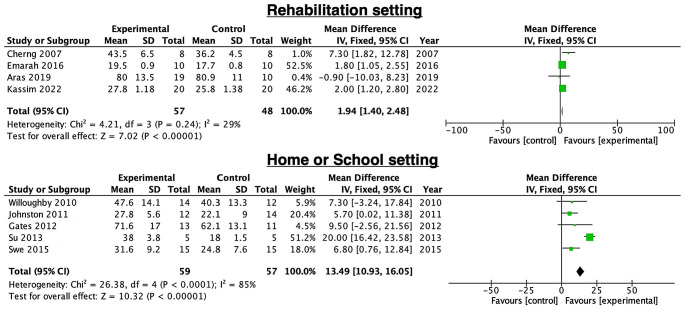
The difference in the effects of PBWSTT between rehabilitation [25,28,31,32], home [26,29], or school setting [3,16,27] in patients with cerebral palsy. The colored squares represent the odds ratio.

**Figure 5:**
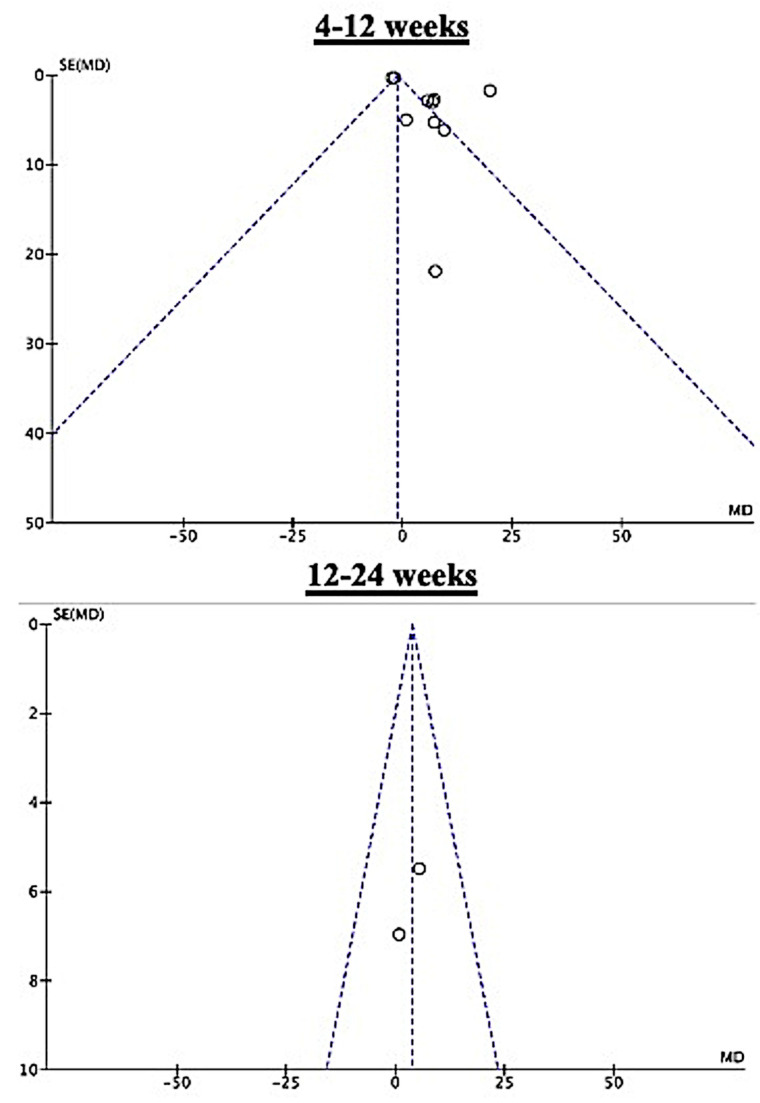
Funnel plot. The funnel is depicted by dashed lines, and the individual studies’ effects are represented by open circles.

Figure 4 compares the effectiveness of PBWSTT in rehabilitation, home, or school setting. The effectiveness of PBWSTT in a rehabilitation context was examined in four trials [25,28,31,32] comprising 105 patients. There was less variation between the studies, but this was not statistically significant (I^2^ = 29%, *p* = 0.24). In a rehabilitation environment, PBWSTT for 4 to 12 weeks was significantly effective (MD = 1.94, 95% CI = 1.40—2.48, *p* < 0.0001). Five trials [3,16,26,27,29], comprising 116 patients, examined the effect of PBWSTT for 4 to 12 weeks, and this was found to be significantly effective in a home or school setting (MD = 13.5, 95% CI = 13.9—16.0, *p* < 0.0001). There was significant heterogeneity between studies (I^2^ = 85%, *p* < 0.0001). The studies [3,16,25,26,27,28,29] in the funnel plot that demonstrate the benefits of 4–12 weeks and 12–24 weeks of PBWSTT were all equally beneficial (Figure 5).

## 4. Discussion

The effectiveness of PBWSTT, particularly in rehabilitation, at-home, or school settings, for children aged 3 to 18 years with CP was assessed for the first time in this study. The evidence from this review demonstrated that 4–12 weeks of PBWSTT was effective, especially in rehabilitation, at-home, or school settings. There were insufficient data to determine the effects of PBWSTT over a period of 12–24 weeks.

In the qualitative synthesis, studies from Turkey [23], Australia [30], and Saudi Arabia [31] indicated that PBWSTT over 4–12 weeks might increase walking speed in children with CP in rehabilitation or school settings. The three other trials, carried out in Taiwan [25], China [3], and Australia [27], demonstrated that PBWSTT exhibited enhanced gross motor skills in identical settings. In an Indian rehabilitation context [32], PBWSTT resulted in a considerable improvement in walking speed, cadence, stride, and gross motor function. Furthermore, three investigations [16,26,29] found no evidence of a significant effect of PBWSTT on these results.

The evidence from this review is consistent with the best evidence that was available in 2019 [34] and earlier [35,36]. The study demonstrated that PBWSTT helps CP children function better and accomplish tasks more effectively. In children suffering from CP, PBWSTT has been documented to improve walking speed in a previous evaluation of 41 trials, including 11 randomized trials [37]. Another review of 24 randomized trials found that PBWSTT was more effective than resistance training at enhancing gait speed in children with CP [38]. Additionally, the outcomes of previous studies revealed that PBWSTT enhances children’s walking ability [28] and improves gross motor skills [3]. A prior systematic review, however, did not conclude that PBWSTT helps children with CP [39]. Possible reasons could be the lack of participants, the individuals’ varying degrees of skill, and the poor quality of the trials.

The results of this review, however, consist of a prior clinical trial that investigated the outcomes of a school-based, twice-weekly PBWSTT program for six weeks on the endurance and gait speed of children with CP [30]. The trial’s findings suggested that PBWSTT would be a good gait training alternative and it might be possible to implement a similar program in a classroom setting. The results from a two-period randomized crossover design with repeated measures study show that PBWSTT improved gross motor skills in low-functioning children and adolescents with nonspastic CP who were enrolled in special schools [3]. However, the results from a different randomized trial carried out at a special-needs school in Singapore revealed that PBWSTT is not more effective than overground training in enhancing the qualities of walking and other functions in children with mild to moderate CP [27]. A possible reason for these contradictory results in a learning environment could be attributed to interventional parameters, such as speed, body weight support, time per session, and the frequency and duration of use of various types of treadmill training. It is currently impossible to speculate on the criteria that might lead to successful results.

The notable strength of this study lies in the fact that all of the studies incorporated were randomized trials, a design which, under similar conditions, is associated with a high level of evidence [40]. However, there are certain limitations to this study. First, there were methodological issues with the studies included in this evaluation, including a high risk of bias brought on by the lack of blinding. Second, PBWSTT is not well established as the most commonly utilized gait speed outcome [31,41], especially for modest clinically relevant differences. Future studies should aim to adopt a consistent methodology and to pinpoint clinically significant changes. Third, in most rehabilitation trials, it is challenging to entirely blind therapists or participants, making a PEDro score of 8 the best possible result for this review. Finally, the reduced sample size and uneven quality of the research make this study limited. Therefore, it is important to interpret these results with caution.

The findings of this research could facilitate multidisciplinary cooperation amongst rehabilitation, health, and education experts in order to tackle the intricacy of variables involved in the application of PBWSTT in various contexts. A pediatrician, physiotherapist, occupational therapist, and speech therapist are examples of an interdisciplinary team that may be included in the early stages of CP treatment [42]. Every member of the team plays a crucial role in providing the affected child with care. The physiotherapist, for instance, assesses and treats muscular tone, strength, and gait (walking) [43]. An occupational therapist evaluates the child’s capacity for self-care and self-help skills, such as feeding and manual dexterity [9]. The child’s ability to communicate, understand speech, and talk is assessed by the speech therapist [44].

## 5. Conclusions

Our research demonstrates that children or adolescents with CP can benefit from 4 to 12 weeks of PBWSTT in a rehabilitation, at-home, or school setting. There is a lack of research to support the long-term effects of this intervention in many circumstances. Therefore, more research and high-quality randomized trials are undoubtedly necessary to establish the advantages and efficacy that back up the continued use of this intervention in these circumstances. Future investigations, particularly randomized trials with large sample numbers, must also include follow-up measures to ascertain whether children with CP will experience any long-lasting and meaningful advantages.

## Figures and Tables

**Table 1:** Characteristic features of included studies.

Study (Year)	Participants *n*	Group, E/C	Sex, M/F	Age, Mean (SD)	CP Type	Setting	Country
Aras et al. (2019) [28]	29	10/19	18/11	9.3 (2.3)	Spastic	Rehabilitation	Turkey
Cherng et al. (2007) [25]	16	8/8	14/2	5 (2.0)	Spastic	Rehabilitation	Taiwan
Dodd and Foley (2007) [30]	14	7/7	10/4	9.0 (2.7)	Spastic	School	Australia
Emarah et al. (2016) [31]	20	10/10	7/13	6.7 (0.6)	Spastic	Rehabilitation	Saudi Arabia
Gates et al. (2012) [26]	26	14/12	7/7	9.5 (2.2)	Spastic	Home	United States
Johnston et al. (2011) [29]	26	14/12	14/12	9.5 (2.2)	Spastic	Home	United States
Kassim et al. (2022) [32]	60	20/40	-	6.3 (2.0)	Spastic	Rehabilitation	India
Su et al. (2013) [3]	10	5/5	8/2	10.9 (2.0)	-	School	China
Swe et al. (2015) [27]	30	15/15	20/10	13.2 (3.4)	-	School	Australia
Willoughby et al. (2010) [16]	26	12/14	15/11	10.7 (3.6)	-	School	Australia

Abbreviations: *n*, a number of participants, E, experimental group; C, control group; F, female; M, male; yr, year; CP, cerebral palsy.

**Table 2:** Intervention and main findings of included studies.

Study (Year)	Intervention	Treatment Time	Assessment	Outcome	Key Findings
Aras et al. (2019) [28]	E: Treadmill with partial body weight support. C: Robotic-assisted treadmill and anti-gravity treadmill exercise.	E: 20 sessions 45 min 2 times/5 days for 4 weeks. C: 20 sessions 45 min 2 times/5 days for 4 weeks.	Baseline and 4 weeks	Walking capacity	PBWSTT showed a positive impact on walking in patients with spastic CP.
Cherng et al. (2007) [25]	E: Treadmill with partial body weight support. C: The regular treatment.	E: 20 min/session, 2–3 sessions/ week, for a total of 12 weeks. C: 2–3 times/week, 30 min/ session.	Baseline and 12 weeks	Gross motor function, and Temporal–distance parameters of gait.	Children with spastic CP experienced improvements in gross motor abilities, a reduction in the percentage of double-limb support, and longer strides after 12 weeks of PBWSTT.
Dodd and Foley (2007) [30]	E: Treadmill with partial body weight support. C: Walking training.	E: 10 m walking test for 6 weeks. C: 10 m walking test for 6 weeks.	Baseline and 6 weeks	Walking speed.	The implementation of PBWSTT appears to be a viable option for improving the walking speed of children with CP and moderate to severe impairments in a school setting.
Emarah et al. (2016) [31]	E: Conventional physiotherapy plus treadmill with partial body weight support. C: Conventional physiotherapy plus treadmill training.	E: Total 36 training sessions for a duration of 30 min, 3 times/week for a total of 12 weeks. C: Treadmill training for 40 min 3 times per week for 3 months.	Baseline and 12 weeks	Optimal postural stability, good balance, and less effort.	Effective postural stability, good balance control, and reduced exertion are all facilitated by PBWSTT, allowing for a safe and efficient walk.
Gates et al. (2012) [26]	E: Treadmill with partial body weight support. C: Strengthening exercise program.	E: 30 min 5 times/week for a total of 10 weeks. C: 30 min 5 times/week times for a total of 10 weeks.	Baseline, 12, and 16 weeks.	Participation, quality of life, self-concept, goal attainment, and satisfaction.	With PBWSTT, assessments of participation, parent evaluation of QOL, goal attainment, and satisfaction may take place in a comparatively short amount of time (12 weeks).
Johnston et al. (2011) [29]	E: Treadmill with partial body weight support. C: Strengthening exercise.	E: Twice daily/5 days a week for 10 weeks. C: Twice daily/5 days a week for 10 weeks.	Baseline and 12 weeks	Gait speed and cadence.	There is no difference of gain in spatiotemporal parameters (such as gait speed and cadence) between PBWSTT and exercise interventions.
Kassim et al. (2022) [32]	E: Body weight support overground training. C: Conventional gait training.	E: 8-week training. C: 8-week training.	Baseline and 8 weeks	Conventional gait training, body weight-supported walking, cadence, stride, and gross motor function.	Gross motor function, cadence, stride, and walking all significantly improved with the PBWSTT.
Su et al. (2013) [3]	E: Treadmill training incorporating partial body weight support. C: Conventional gait training.	E: 14 sessions with an average of 262 min/12 weeks. C: 16 sessions with an average of 295 min/12 weeks.	Baseline and 12 weeks	Gross motor skills for low functioning	Children and adolescents with nonspastic CP who are low functioning can benefit from PBWSTT in terms of their gross motor abilities.
Swe et al. (2015) [27]	E: Treadmill training incorporating partial body weight support. C: Above the ground.	E: 2 times 30 min/week for 8 weeks C: 2 times 30 min/week for 8 weeks	Baseline, 4 weeks, and 8 weeks	Walking and gross motor functions	After eight weeks of PBWSTT, children with mild to moderate CP had improved gross motor functions and walking speed.
Willoughby et al. (2010) [16]	E: Treadmill training incorporating partial body weight support. C: Walking.	E: Twice/week for 9 weeks. C: Twice/week for 9 weeks.	Baseline, 10, and 24 weeks.	Walking speed and endurance.	Children with CP can no longer improve their walking speed and endurance with 9 weeks of PBWSTT.

Abbreviations: E, experimental group; C, control group, CP, cerebral palsy, PBWSTT, partial body weight-supported treadmill training.

## Data Availability

The data presented in this study are available on request from the corresponding author. The data are not publicly available due to their containing information that could compromise the privacy of research participants.
